# Non-Proteinuric Diabetic Kidney Disease: A Comprehensive Review

**DOI:** 10.3390/life16040533

**Published:** 2026-03-24

**Authors:** Piruthiviraj Natarajan, Fahmida Shaik, Arunita Chatterjee, Sharma S. Prabhakar

**Affiliations:** Department of Medicine, Texas Tech University Health Sciences Center, Lubbock, TX 79430, USA

**Keywords:** diabetes mellitus, proteinuria, chronic kidney disease, nephropathy

## Abstract

Diabetic kidney disease (DKD) persists as the leading cause of chronic kidney disease (CKD) and often leads to end-stage renal disease (ESRD). Worldwide, 30–50% of patients with diabetes are affected by DKD, while DKD contributes to about half of ESRD. Previously, DKD had been defined based on overt proteinuria—that is, a urine albumin-to-creatinine ratio (UACR) above 300 mg/g—after a stage of microalbuminuria (UACR 30–300 mg/g). However, emerging data suggest that a significant number of patients develop renal functional decline without albuminuria, suggesting that DKD can occur in the absence of protein excretion. This phenotype of normoalbuminuric or non-proteinuric DKD (NA-DKD or NP-DKD) is emerging as an important clinical entity. It is characterized by a gradual decline in renal function, commonly with an annual reduction in estimated glomerular filtration rate (eGFR) > 3 mL/min/1.73 m^2^ or an eGFR < 60 mL/min/1.73 m^2^, while the UACR remains < 30 mg/g. Growing rates of NP-DKD expose limitations inherent in traditional models of DKD pathogenesis and underscore the need for diagnostic and therapeutic paradigms that are not reliant on albuminuria-only criteria. Here, we present a comprehensive review of the NP-DKD to guide a more inclusive model of DKD pathogenesis, its diagnosis, and therapy.

## 1. Introduction

DKD is a serious public health problem with huge morbidity, mortality, and healthcare burden. Traditionally, albuminuria has been viewed as the “gold standard” feature of DKD, serving as a surrogate marker of diagnosis and disease progression. Sensitivity assays for microalbuminuria made early detection of renal injury possible. Yet, increasing evidence is now available to recognize that a substantial number of diabetic subjects with low eGFR never demonstrated albuminuria (UACR < 30 mg/g), thus suggesting a separate pathophysiology [1,2].

The emergence of normoalbuminuric DKD warranted re-evaluation of the traditional linear model of disease progression. Historically, DKD was considered to progress from glomerular hyperfiltration through the development of microalbuminuria to overt proteinuria, declining eGFR, and ultimately ESRD. Recent studies have shown that a non-proteinuric course is observed in as many as 40% of patients, especially if they are affected by type 2 diabetes [3,4]. These results indicate that the loss of kidney function in diabetes may be driven by a mechanism independent of the glomerular injury–proteinuria pathway.

There are two important consequences of this insight. First, screening for diabetes-induced renal functional decline based on albuminuria alone can miss a substantial number of patients at risk. Secondly, NP-DKD likely represents a distinct pathophysiologic entity leading to renal failure, with greater renal vascular injury, tubulointerstitial injury, and inflammation involved in its progression compared with traditional diabetic glomerulopathy, which is caused primarily by glomerular damage [5].

The entity NP-DKD has been described variably in the literature as non-albuminuric renal disease, normoalbuminuric diabetic nephropathy, non-proteinuric diabetic kidney disease, or normoalbuminuric diabetic renal insufficiency [2]. All these terms refer to the same heterogeneous condition: renal functional decline without albuminuria, observed in patients with diabetes. In this article, we have used NA-DKD and NP-DKD, but they refer to the same condition.

## 2. Clinical Course

Conventional DKD has a progression path from hyperfiltration to microalbuminuria and to overt proteinuria, as well as deteriorating kidney function. NP-DKD, on the other hand, features renal functional deterioration in the absence of preceding albuminuria, as already stated. The prevalence of NP-DKD varies from 20 to 40% in general, representing about 20% of subjects with type 1 diabetes and up to 40% of those with type 2 diabetes [3,4]. As with most diseases, including DKD, the prevalence of NP-DKD can vary widely depending on age distribution, diabetes duration, glycemic control, ethnicity, eGFR calculation methods, and medication use.

The pathogenesis of NP-DKD remains complex. Associating factors are older age, hypertension, dyslipidemia, obesity, hyperuricemia, and chronic microangiopathy. Use of reno-protective agents, like renin–angiotensin–aldosterone system (RAAS) blockers, can influence development as well, for obvious reasons [2]. The phenotype is more frequent in older individuals (typically aged 60–79 years), raising the question of whether age-related vascular changes or diabetic microvascular damage are involved in its pathophysiology.

Female sex is an independent risk factor of NP-DKD, which has been revealed by epidemiological studies. Sangha et al. reported that NP-DKD patients were older than proteinuric DKD subjects (56.5 ± 2.1 vs. 54.7 ± 11.6 years), had a lower prevalence of diabetic retinopathy (46.2% vs. 74.1%), and had higher levels of hemoglobin and total cholesterol [6]. Similarly, in a review by Fabre et al. NP-DKD was noted to be associated with female sex, less diabetic retinopathy (38.9% vs. 66.4%), more favorable renal outcomes, and higher serum albumin levels compared with proteinuric DKD [7]. These patients also had lower LDL cholesterol and a slight decrease in HDL cholesterol levels.

In a Korean study of 562 people with type 2 diabetes, normoalbuminuric DKD was more common in women, subjects with shorter duration of diabetes, and patients taking antihypertensive drugs less frequently than the albuminuric DKD [8]. The incidence of non-albuminuric renal impairment decreased with longer duration of diabetes and more severe retinopathy [9], confirming that older age, female sex, and fewer microvascular complications are characteristics of NP-DKD.

Clinically, patients with NP-DKD generally exhibit a more favorable metabolic and cardiovascular profile than those with albuminuric renal impairment. BMI, systolic blood pressure, cholesterol levels, and the incidence of retinopathy were found to be lower, even though HbA1c was higher, in patients with NP-DKD than in patients with proteinuric DKD [5]. Nevertheless, the association between RAAS blockade and NP-DKD is uncertain. A role for RAAS inhibitors may be indicated. Increased use of RAAS inhibitors in NA-DKD patients could potentially attenuate or eliminate proteinuria; however, most studies report a decreased use of RAAS inhibitors in NP-DKD cohorts [9,10,11].

Blood pressure regulation also seems more effective in NP-DKD, even with less frequent administration of RAAS inhibitors [11]. In a multicenter biopsy-based cohort study, NP-DKD patients featured reduced systolic and diastolic pressures, better lipid control (lower cholesterol levels, more frequent statin use), and better 5-year CKD progression-free survival (86.6 vs. 30.3%) as well as greater degrees of death-free survival (98.4 vs. 87.5% at years) compared to proteinuric DKD [5]. These results suggest that proteinuria is still a powerful determinant of poor renal outcomes and death.

Overall, the risk of cardiovascular disease and other complications remains high in patients with NP-DKD [7]. In a large cohort study, Penno et al. observed that NP-DKD was associated with a lower prevalence of cardiovascular disease than albuminuric DKD; however, the prevalence was higher in NP-DKD than in albuminuria without eGFR reduction [9]. Studies reporting a similar or increased risk of cardiovascular disease in NP-DKD also exist. Yamanouchi et al. reported fewer renal events and lower all-cause mortality in NP-DKD than in proteinuric DKD; however, they did not examine cardiovascular events [5]. While the cardiovascular risk profile of NP-DKD has been variably reported, most studies observe a lower risk in NP-DKD patients compared with proteinuric DKD. However, further studies with larger populations are required to more clearly assess cardiovascular disease risk in NP-DKD. If confirmed, the comparatively higher risk in proteinuric DKD is plausible, as proteinuria and eGFR reduction are both widely accepted as very strong and independent risk factors for cardiovascular morbidity and mortality.

Biophysical characteristics further vary with the phenotype. The renal resistivity index (RRI), a possible indicator of tubulointerstitial damage, is elevated in NP-DKD and indicates a leading association with the tubular system and vessel units [12,13].

## 3. Pathogenesis

The pathogenesis of diabetic kidney disease (DKD) is multifactorial, implicating metabolic derangements, hemodynamic changes, and inflammatory processes. Long-term hyperglycemia causes structural and functional damage to the glomerulus—specifically to the podocytes—(resulting in thickening of the glomerular basement membrane, mesangial expansion, and finally albuminuria) and tubulo-interstitium leading to progressive decline in eGFR [14].

**Figure 1:**
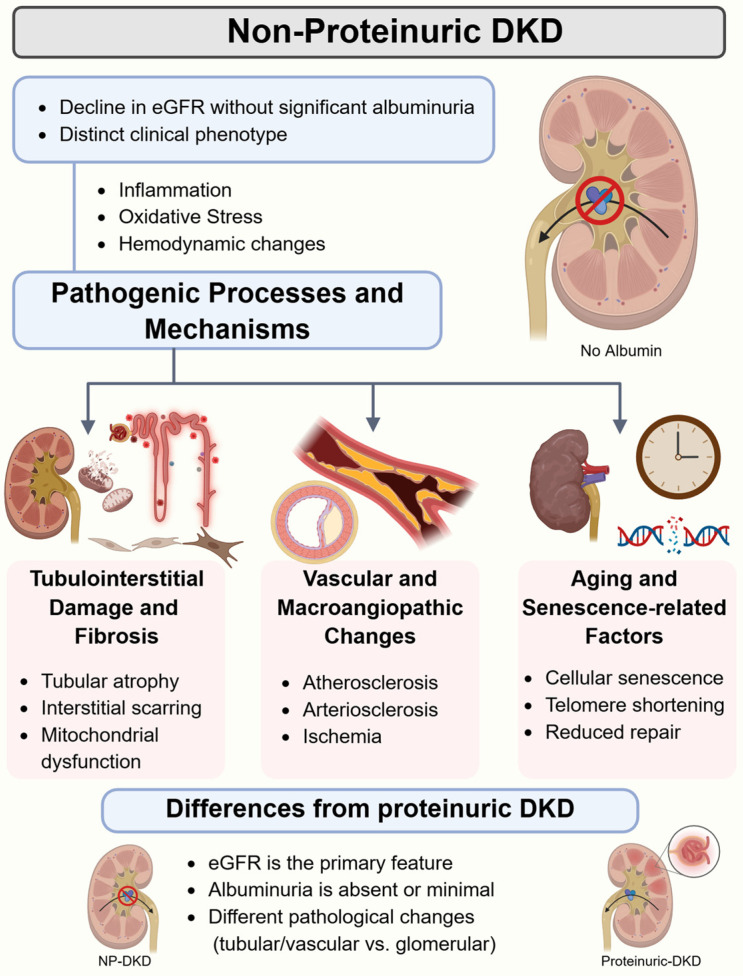
An overview of non-proteinuric diabetic kidney disease (NP-DKD) pathogenesis. eGFR: estimated glomerular filtration rate. Created in BioRender. Chatterjee, A. (2026) https://BioRender.com/n17yuws (accessed on 17 March 2026).

However, NP-DKD seemingly takes another route in pathophysiology. It has been reported that although most albuminuric DKD cases show a characteristic glomerulopathy, roughly half of the NP-DKD patients do not show this lesion but rather arteriosclerosis and tubulointerstitial fibrosis at variable magnitudes [12]. These results strongly indicate separate mechanisms for both phenotypes (Figure 1).

### 3.1. Tubulointerstitial and Vascular Injury

Histological studies suggest that NP-DKD reflects vascular and tubulointerstitial, rather than glomerular, disease. Indeed, in biopsy series from Japan and Australia, nearly 60% of NP-DKD patients had glomeruli proximal to normalcy while having advanced arteriosclerosis and interstitial fibrosis [5,12]. This is consistent with the idea that NP-DKD represents a macroangiopathic process, secondary to systemic vascular disease, as opposed to the microangiopathy present in proteinuric DKD [15].

### 3.2. Recurrent Subclinical Acute Kidney Injury (AKI)

One of the emerging experimental hypotheses is that NP-DKD may be caused by repeated, albeit often only subclinical, episodes of acute kidney injury. These episodes could be ischemic, infectious, or toxic and progressively result in tubular atrophy and interstitial fibrosis [16]. Diabetic subjects with such insults are particularly susceptible to severe renal injury as a result of tubular hypoxia, diminished regeneration, and RAAS-blocker-induced hemodynamics [9,17]. Eventually, these pathogenic triggers culminate in disease progression, leading to a decline in eGFR without albuminuria.

### 3.3. Arteriolar Disease and Endothelial Dysfunction

Endothelial dysfunction and vascular remodeling are hallmarks of NP-DKD. Renal perfusion and oxygen delivery are compromised by hyaline arteriolosclerosis, medial hypertrophy, and luminal narrowing [18]. Such hypoxic injury leads to activation of profibrotic processes through hypoxia inducible factor-1-alpha (HIF-1α), transforming growth factor-beta (TGF-β)**,** and nuclear factor kappa-B (NF-kB) signaling pathways [19]. These factors enhance interstitial fibrosis and tubular damage, which are independent of glomerular injury.

### 3.4. Macroangiopathy Versus Microangiopathy

There are several papers that support the role of macroangiopathy (as opposed to microangiopathy) as a primary pathogenic initiator in NP-DKD. This notion is also supported by the less frequent association of NP-DKD with diabetic retinopathy when compared with proteinuric DKD, as previously reported [8,9]. NP-DKD patients demonstrate increased arterial stiffness and higher carotid intima-media thickness, consistent with systemic atherosclerotic load [4,9,20].

### 3.5. Tubular Albumin Handling

Preservation of tubular albumin reabsorption is an additional proposed mechanism. An intact tubular reuptake system (in particular, the megalin–cubilin complex) may resist albuminuria despite having glomerular damage. Increased reabsorption may thus protect against the development of nephrosis by sustaining the normoalbuminuric status. Moreover, increased protein accumulation in tubular cells could induce inflammation, thereby accounting for the higher tubulointerstitial injury observed in NP-DKD patients.

### 3.6. Inflammatory and Molecular Pathways

Inflammation and fibrosis are prominently involved in NP-DKD. Biomarkers (e.g., neutrophil gelatinase-associated lipocalin (NGAL), kidney injury molecule-1 (KIM-1), and liver-type fatty acid-binding protein (L-FABP)), which signal tubular stress, are associated with progression of disease [21]. High circulating levels of TNF and Fas ligand provide evidence of markers of systemic and renal inflammatory activation. Macrovascular inflammation promotes an additional contribution of renal hypoxia and fibrosis [14].

**Figure 2:**
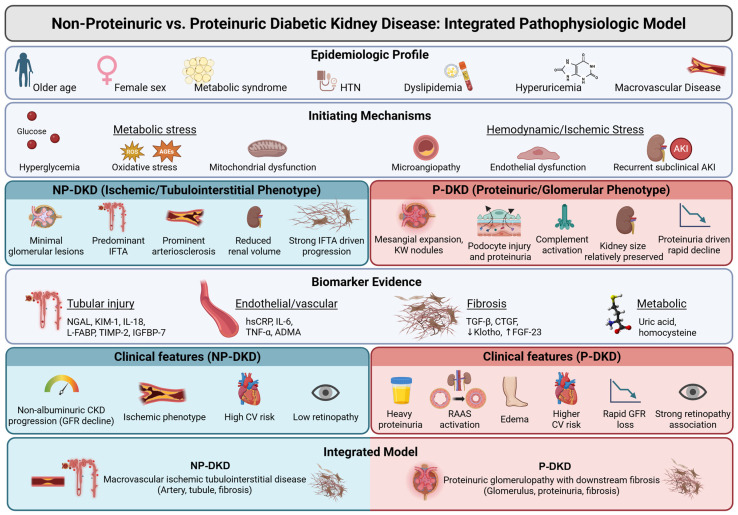
An integrated pathophysiologic model comparing non-proteinuric and proteinuric DKD. Arrows (upward or downward) indicate the expected change in biomarker levels (increase or decrease). HTN: hypertension, AKI: acute kidney injury, ROS: reactive oxygen species, AGEs: advanced glycation end-products, IFTA: interstitial fibrosis and tubular atrophy, NGAL: neutrophil gelatinase-associated lipocalin, KIM-1: kidney injury molecule 1, IL: interleukin, L-FABP: liver-type fatty acid–binding protein, TIMP-2: tissue inhibitor of metalloproteinases 2, IGFBP-7: insulin-like growth factor-binding protein 7, hsCRP: high-sensitivity C-reactive protein, TNF-α: tumor necrosis factor alpha, ADMA: asymmetric dimethylarginine, TGF-β: transforming growth factor-beta, CTGF: connective tissue growth factor, FGF-23: fibroblast growth factor 23, CKD: chronic kidney disease, GFR: glomerular filtration rate, CV: cardiovascular, RAAS: renin–angiotensin–aldosterone system, NP-DKD: non-proteinuric diabetic kidney disease, P-DKD: proteinuric diabetic kidney disease. Created in BioRender. Chatterjee, A. (2026) https://BioRender.com/4fk8oxb (accessed on 17 March 2026).

Together, these observations underline that NP-DKD is not a “milder” form of proteinuric DKD, but rather behaves as a different phenotype characterized by predominant vascular and tubulointerstitial damage, with relatively limited glomerular injury. A summary of these pathogenic pathways in NPDKD is presented in Figure 2.

## 4. Renal Pathology

Key histopathological features separating chronic kidney disease (CKD) with proteinuria from NP-DKD are shown in comparative renal biopsy studies. In NP-DKD, less severe glomerular lesions with less mesangial expansion and fewer Kimmelstiel–Wilson nodules are found [14]. On the other hand, tubulointerstitial and vascular changes—especially interstitial fibrosis, tubular atrophy, and arteriosclerosis—are more apparent [5].

In a retrospective Japanese cohort of approximately 396 patients, glomerular lesions were strong determinants of renal outcomes in proteinuric diabetic kidney disease; however, they were not independent predictors of progression to end-stage renal disease in non-proteinuric DKD, in which interstitial fibrosis and tubular atrophy emerged as the principal determinants of disease progression [5]. While diabetic glomerulopathy represents the characteristic histopathological pattern of DKD [22], subsequent biopsy-based analyses demonstrated that nearly all albuminuric DKD patients exhibit classic glomerular lesions, whereas only about half of NP-DKD patients do so, with arteriolosclerosis being universally present in NP-DKD [12].

In addition, immunofluorescence findings differ between the phenotypes. NP-DKD patients show much lower deposition of immunoglobulin M (IgM) and complement components C1q and C3 as compared to proteinuric DKD [12]. Activation of the complement system is associated with greater structural damage and functional impairment [23]. Interstitial fibrosis and tubular atrophy (IFTA) are more prominent in NP-DKD and point to IFTA as a potentially more robust prognostic marker than glomerular lesions, and that disease progression is driven primarily by the tubulointerstitial component.

## 5. Diagnosis

DKD is typically diagnosed in a diabetic patient based on albuminuria and/or reduced eGFR after excluding other renal diseases. Yet, the remarkable lack of albuminuria in NP-DKD challenges this diagnostic paradigm. Almost half of patients with diabetes and abnormal renal function do not have albuminuria. Although eGFR is still an important parameter, it can be limited by muscle mass, diet, or assay variability [24]. NP-DKD is therefore underrecognized in a high proportion of cases when clinicians use the clinical practice of identifying DKD only on albuminuria or creatinine-based eGFR.

Due to these limitations, new biomarkers have emerged that may enable early diagnosis and risk stratification. The most predictive biomarkers that are easily measured in routine clinical settings are NGAL, cystatin C, VEGF, and L-FABP [21].

Neutrophil gelatinase-associated lipocalin (NGAL) is secreted by damaged proximal tubular cells and is an early indicator of ischemic tubular injury. Urinary NGAL is an early marker of kidney damage before the development of albuminuria [25].

Cystatin C, freely filtered at the glomerulus and produced at a constant rate by all nucleated cells, is less influenced by muscle mass or diet. As per KDIGO guidelines, cystatin C measurement is recommended for confirmation in patients with eGFR of 45–59 mL/min/1.73 m^2^ based on creatinine formulae (KDIGO 2021 [26]). In NP-DKD, high cystatin C values are associated with both poor diagnosis and prognosis [27].

Liver-type fatty acid-binding protein (L-FABP) correlates with tubular oxidative stress and inflammation. Compared with healthy controls, urinary L-FABP concentrations are higher in NP-DKD and associated with the severity of the disease [28].

Vascular endothelial growth factor (VEGF) secreted by podocytes in early DKD induces glomerular permeability and promotes hyperfiltration. Elevated urinary VEGF levels are found in normoalbuminuric type 2 diabetes and are associated with albuminuria and disease progression [29].

While some emerging biomarkers, such as KIM-1, Netrin-1, urinary microRNAs (miR-21, miR-124), and connective tissue growth factor (CTGF), are promising in terms of utility and clinical use, the potential of these emerging biomarkers needs to be validated in studies with large sample sizes and longer follow-up times before being considered for clinical use [21].

## 6. Treatment

The principles of management of non-proteinuric DKD (NP-DKD) are similar to those for DKD with proteinuria, but the main areas of focus are renoprotection, cardiovascular risk reduction, and the optimization of glycemia and BP. Nonetheless, as pathophysiological mechanisms of NP-DKD differ from the classic DKD paradigm and as most renoprotective trials targeted albuminuric patients, leading to less robust evidence in NP-DKD, newer insights suggest that therapy may be tailored more specifically towards vascular/tubulointerstitial injury as opposed to glomerular injury and proteinuria.

### 6.1. Current Standard of Care

#### 6.1.1. Glycemic Control

The basis of DKD management continues to be optimal glycemic control. Virtually all landmark trials, such as the UK Prospective Diabetes Study (UKPDS) and the Diabetes Control and Complications Trial (DCCT), show that sustained reduction in HbA1c translates into significant reductions in the incidence of microvascular complications, including nephropathy (UKPDS Group, 1998 [30]; DCCT Research Group, 1993 [31]). These renal advantages may be mediated through enhanced structural and functional integrity of both the vasculature and the tubular–interstitial regions in addition to glomerular protection [9].

#### 6.1.2. Blood Pressure Management

Maintaining strict blood pressure control is important for preserving renal function and minimizing cardiovascular morbidity. According to the Kidney Disease: Improving Global Outcomes (KDIGO) 2021 guidelines, the new recommendation for CKD in diabetic patients, regardless of albuminuria status, is to target a systolic blood pressure < 120 mmHg (if tolerated) (KDIGO, 2021 [26]). Notably, patients with NP-DKD usually have better blood pressure control, despite less frequent use of renin–angiotensin–aldosterone system (RAAS) inhibitors [5]. This observation implies distinct hemodynamic properties, perhaps less glomerular hypertension in NP-DKD than in the proteinuric variant.

#### 6.1.3. RAAS Blockade

ACEi and ARBs are standard therapy for the management of albuminuric DKD, since both classes have antiproteinuric and renoprotective benefits. Use of RAAS inhibitors reduces proteinuria, as observed in previous studies [32]. Therefore, greater use of RAAS inhibitors among NP-DKD patients could explain the absence of proteinuria; however, in most reports, less frequent use of RAAS inhibitors has been noted in NP-DKD patients [9,10,11]. However, given the systemic benefits of RAAS blockade, it is still reasonable to study this aspect further, and indeed it seems quite strongly indicated to use these agents in NP-DKD patients with hypertension or cardiovascular comorbidities.

#### 6.1.4. Lifestyle and Risk Factor Modification

For both phenotypes, lifestyle modification is still important. Weight loss, sodium restriction, smoking cessation, and particularly control of lipids with statins are cornerstones in the reduction of cardiovascular and renal risk [9]. While NP-DKD patients are better protected at the metabolic and lipid levels than those with proteinuric DKD, their cardiovascular risk remains considerable and warrants a holistic approach to cardiovascular risk reduction.

### 6.2. Newer Therapies

#### 6.2.1. SGLT2 Inhibitors

Sodium-glucose cotransporter 2 (SGLT2) inhibitors, including empagliflozin, dapagliflozin, and canagliflozin, have profoundly altered the outcomes of DKD by delaying CKD progression and decreasing cardiovascular events, effects which remain observable in patients with little or no albuminuria [33]. In these same post hoc analyses, the renoprotective effect of SGLT2 inhibitors was confirmed among normoalbuminuric diabetic patients, probably due to renal hemodynamic and anti-inflammatory effects, which are independent of glycemic control.

#### 6.2.2. Non-Steroidal Mineralocorticoid Receptor Antagonists (MRAs)

Finerenone is a selective non-steroidal MRA that has shown substantial reductions in kidney failure and cardiovascular end points among diabetic CKD populations in the FIDELIO-DKD and FIGARO-DKD trials [34]. While these studies enrolled mainly albuminuric patients, the pathways that the finerenone targets—inflammation and fibrosis—are also central to NP-DKD pathogenesis and may be a potential therapeutic target.

#### 6.2.3. GLP-1 Receptor Agonists

Glucagon-like peptide-1 receptor agonists (GLP-1RAs) (liraglutide, semaglutide, dulaglutide) improve cardiovascular outcomes and may protect the kidney by various mechanisms, including reductions in blood pressure and body weight, and anti-inflammatory effects [35]. While data on renal outcomes are better established in albuminuric DKD, emerging evidence suggests benefits across all phenotypes, including NP-DKD.

#### 6.2.4. Endothelin Receptor Antagonists (ERAs)

Endothelin antagonists may have a direct antiproteinuric and renoprotective effect in diabetic nephropathy, as already demonstrated with agents such as atrasentan and sparsentan [36]. The potential benefit of these vascular and inflammation-modifying mechanisms in NP-DKD remains to be explored, but could be especially relevant given the abundance of arteriosclerotic and endothelial injury in this phenotype.

### 6.3. Future Directions

#### 6.3.1. Biomarker-Guided Therapy

Reliance on albuminuria for diagnosis and monitoring has been inadequate for the management of NP-DKD. Emerging biomarkers, such as NGAL, KIM-1, cystatin C, L-FABP, and VEGF, may enable earlier identification, stratification, and personalization of therapy [21]. Next, trials featuring biomarker-based treatment methodology may improve risk prediction and patient selection for therapy.

#### 6.3.2. Anti-Inflammatory and Anti-Fibrotic Approaches

Targeting inflammation and fibrosis, because they play such prominent roles in NP-DKD, is a rational therapeutic pathway in this context. Renoprotective agents are currently under investigation, including pentoxifylline, pirfenidone, and connective tissue growth factor (CTGF) inhibitors. Furthermore, inhibition of cytokine cascades, like TNF or TGF-β, may potentially attenuate tubulointerstitial injury [37].

#### 6.3.3. AKI-to-CKD Transition Prevention

The idea that NP-DKD develops via repeated episodes of undetected acute kidney injury suggests that prevention would be optimal and should target these events. Identification of high-risk patients and limiting nephrotoxic exposures while in hospital, during surgery, or during contrast studies might help to prevent the transition from AKI to CKD [37].

#### 6.3.4. Precision Medicine Approaches

Precision medicine is a rapidly evolving field of tailoring therapy to a patient’s unique characteristics. In addition to an accurate diagnosis and following specific prognostic biomarkers, precision medicine approaches require refining therapeutic strategies to account for an individual’s genetic makeup, lifestyle, and environmental factors. While genomics, proteomics, and metabolomics have been explored in the heterogeneous spectrum of DKD diagnosis and prognoses (reviewed by Downie et al., 2023 [38]), specific examples of their use in NPDKD remain limited. However, improved NP-DKD classification and management using multi-omics profiling, renal imaging (e.g., vascular stiffness, renal volume), and histologic phenotyping may turn out to be novel approaches. Targeting the implicated pathogenic pathways may thus differentiate macroangiopathic from microangiopathic processes, which would allow combining therapies for vascular protection with metabolic control [5,15]. However, thorough research aimed at filling knowledge gaps regarding the variability in the pathogenesis of NP-DKD across populations is a prerequisite. We have a long way to go, but the future seems bright.

## 7. Outcomes

Compared with classic proteinuric DKD, NP-DKD has different clinical outcomes. NP-DKD patients have a reduced risk for ESRD progression, but cardiovascular morbidity is high. In addition, there are differences in the rate of progression of renal failure between the two types of DKD. In a prospective observational study of 400 patients with type 2 diabetes, 26.5% met criteria for NP-DKD. Compared with proteinuric DKD, these patients had higher eGFR at baseline, 6 months, and 1 year, suggesting a slower rate of renal decline [6].

Extra-renal implications of NP-DKD are also of great importance. According to the International Working Group on the Diabetic Foot (IWGDF) (2016) [39] and National Institute for Health and Care Excellence (NICE) (2023) [40], diabetic kidney disease (DKD) is a risk factor for the development of diabetic foot ulcers, amputation, and mortality. Since screening for DKD is still associated with proteinuria, NP-DKD cases might go unnoticed in the early stages, necessitating greater vigilance when screening diabetic patients with decreasing eGFR but normal UACR.

## 8. Conclusions

Non-proteinuric diabetic kidney disease constitutes an important, distinct, and common phenotype of diabetic nephropathy. Unlike classic diabetic kidney disease (DKD), which is caused by the leakage of proteins from the glomeruli leading to progressive albuminuria, NP-DKD is caused by vascular and tubulointerstitial mechanisms, and often occurs without evidence of glomerular pathology. Clinically, compared with patients with type 2 diabetes mellitus (T2DM) and DKD, NP-DKD patients are older, more frequently female, and more often have microvascular complications such as retinopathy. Cardiovascular morbidity and mortality are significant despite the generally better renal prognosis compared with proteinuric DKD. Tools that solely quantify albuminuria may underestimate disease prevalence and underscore the relevance of a biomarker-driven approach that enables more tailored management. Newer therapies like SGLT2 inhibitors, non-steroidal MRAs, and GLP-1 receptor agonists are likely to slow the progression of both phenotypes. Future studies will need to address molecular mechanisms, validate diagnostic biomarkers, and personalize management of vascular-related renal injury. The acknowledgment of NP-DKD as an independent clinical and pathophysiological entity represents a paradigm shift in the understanding and management of DKD.

## Data Availability

No new data were created or analyzed in this study. Data sharing is not applicable to this article.

## Abbreviations

The following abbreviations are used in this manuscript:

ACEi	Angiotensin-converting enzyme inhibitors
ADMA	Asymmetric dimethylarginine
AGEs	Advanced glycation end-products
AKI	Acute kidney injury
ARBs	Angiotensin receptor blockers
BMI	Body mass index
CKD	Chronic kidney disease
CTGF	Connective tissue growth factor
DKD	Diabetic kidney disease
eGFR	Estimated glomerular filtration rate
ERAs	Endothelin receptor antagonists
ESRD	End-stage renal disease
FGF-23	Fibroblast growth factor 23
GLP-1	Glucagon-like peptide-1
GLP1-RA	Glucagon-like peptide-1 receptor agonists
HbA1c	Hemoglobin A1c
HDL	High-density lipoprotein
HIF-1α	Hypoxia inducible factor-1-alpha
hsCRP	High-sensitivity C-reactive protein
HTN	Hypertension
IFTA	Interstitial fibrosis and tubular atrophy
IGFBP-7	Insulin-like growth factor-binding protein 7
IgM	Immunoglobulin M
KIM-1	Kidney injury molecule-1
L-FABP	Liver-type fatty acid–binding protein
LDL	Low-density lipoprotein
MRAs	Mineralocorticoid receptor antagonists
NA-DKD	Normoalbuminuric diabetic kidney disease
NF-kB	Nuclear factor kappa-B
NGAL	Neutrophil gelatinase-associated lipocalin
NP-DKD	Non-proteinuric diabetic kidney disease
P-DKD	Proteinuric diabetic kidney disease
RAAS	Renin–angiotensin–aldosterone system
ROS	Reactive oxygen species
RRI	Renal resistivity index
SGLT2	Sodium-glucose cotransporter 2
T2DM	Type 2 diabetes mellitus
TIMP-2	Tissue inhibitor of metalloproteinases 2
TGF-β	Transforming growth factor-beta
TNF	Tumor necrosis factor
UACR	Urine albumin-to-creatinine ratio
VEGF	Vascular endothelial growth factor
